# Targeting vivax malaria in the Asia Pacific: The Asia Pacific Malaria Elimination Network Vivax Working Group

**DOI:** 10.1186/s12936-015-0958-y

**Published:** 2015-12-01

**Authors:** The Vivax Working Group

**Affiliations:** The APMEN Vivax Working Group, Global and Tropical Health Division, Menzies School of Health Research and Charles Darwin University, Darwin, NT 0810 Australia

**Keywords:** Malaria, *Plasmodium vivax*, APMEN, Asia-Pacific, Elimination

## Abstract

The Asia Pacific Malaria Elimination Network (APMEN) is a collaboration of 18 country partners committed to eliminating malaria from within their borders. Over the past 5 years, APMEN has helped to build the knowledge, tools and in-country technical expertise required to attain this goal. At its inaugural meeting in Brisbane in 2009, *Plasmodium vivax* infections were identified across the region as a common threat to this ambitious programme; the APMEN Vivax Working Group was established to tackle specifically this issue. The Working Group developed a four-stage strategy to identify knowledge gaps, build regional consensus on shared priorities, generate evidence and change practice to optimize malaria elimination activities. This case study describes the issues faced and the solutions found in developing this robust strategic partnership between national programmes and research partners within the Working Group. The success of the approach adopted by the group may facilitate similar applications in other regions seeking to deploy evidence-based policy and practice.

**Electronic supplementary material:**

The online version of this article (doi:10.1186/s12936-015-0958-y) contains supplementary material, which is available to authorized users.

## Background

### The importance of *Plasmodium vivax* in elimination

Once regarded as a relatively benign infection, *Plasmodium vivax* is now acknowledged to be an important public health threat, capable of causing life-threatening disease complications, debilitating recurrent infections, miscarriage and chronic infections [1–6]. The increasing sensitivity of diagnostic tools [7] has significantly improved the understanding of current *P. vivax* epidemiology and it has become apparent that the true burden of vivax malaria infections is significantly higher than previously assumed [8]. Furthermore, although major gains have been made in the reduction of malaria over the last decade [9], in almost all co-endemic regions these successes are far greater for *Plasmodium falciparum* than for *P. vivax*. Compared with *P. falciparum, P. vivax* exhibits far more extensive genetic diversity [10–13] and has numerous adaptive biological mechanisms, such as the ability to develop dormant liver stages (hypnozoites) and the emergence of transmissible blood stages (gametocytes) before clinical symptoms [14]. These properties afford the parasite a variety of strategies to adapt to environmental challenges, including those imposed by intensive malaria control activities.

The adaptability of *P. vivax* makes it difficult to contain and highly prone to resurgence especially when control efforts cannot be sustained [15]. Hence, treating all stages of the parasite (radical cure) is a critical strategy for the successful control and ultimate elimination of *P. vivax*. Radical cure of *P. vivax* requires clearance of blood stage parasites as well as the hypnozoites, which result in relapse and re-establishment of a blood stage infection. Chloroquine has remained the preferred treatment for *P. vivax* blood stage infections in most endemic countries, however this policy is under threat from emerging drug resistant *P. vivax* strains [16]. The only current widely available drug with activity against hypnozoites is the 8-aminoquinoline compound, primaquine. Unfortunately, individuals who have a genetic deficiency in the enzyme glucose-6-phosphate dehydrogenase (G6PD) are at risk of severe haemolysis when treated with the drug [17]. In addition, primaquine requires prolonged daily administration over seven to 14 days. The complexities of prescribing reliable, safe and effective radical cure of *P. vivax* highlights the urgent need for innovative new approaches to assure schizonticidal and hypnozoiticidal treatment; without both activities, *P. vivax* elimination is unnecessarily delayed or unlikely in most settings.

### APMEN history

The Asia Pacific Malaria Elimination Network (APMEN) was established in 2009 to create an innovative, country-led platform to support malaria elimination in the Asia Pacific region [18, 19]. APMEN brings together a wide range of stakeholders from across the region to support each other to achieve individual country targets and the long term collective goal of regional and supra-regional malaria elimination [20, 21]. In November 2014 the East Asia Summit in Yangon, attended by 18 country representatives, collectively committed to the elimination of malaria from the Asia Pacific by 2030 [22]. The achievement of such a milestone will require substantial and sustained political and financial commitment, as well as the implementation of innovative and broad reaching malaria control strategies. The established APMEN Vivax Working Group (VxWG) provides a unique forum for the interdisciplinary collaboration necessary to achieve this, generating evidence based strategies targeting *P. vivax* that can be translated rapidly into policy and practice [23]. In this article the development, activities and achievements of the VxWG are reviewed, and its goals, strategic processes, governance, and future direction are presented as a model for collaborative malaria engagement.

### Establishment of the APMEN Vivax Working Group

The VxWG was established at the inaugural meeting of APMEN in 2009. The participants at this meeting included 10 National Control Programmes, numerous scientific and academic institutions, the World Health Organization (WHO), and the Australian Government. These founding members unanimously agreed that *P. vivax* posed a considerable challenge to malaria elimination in the Asia Pacific region, due mainly to the paucity of evidence required from which appropriate control strategies can be devised and implemented [21]. The APMEN VxWG was established with the mandate to develop and coordinate an operational research agenda to fill this gap [24–26] and to provide the evidence base for national and regional policy makers. The greatest burden of *P. vivax* is in infants and pregnant women [5, 27]. Therefore, the VxWG agenda had direct relevance to achieving the Millennium Development Goals 4, 5 and 6 current at that time. The agenda was also aligned with Roll Back Malaria (RBM) Global Malaria Action Plan (2010–2015), which encourages support of countries pursuing elimination through collection and dissemination of best-practice approaches, research and development for new tools, and provision of funding and technical assistance by partners. The recently released Global Technical Strategy for Malaria 2016–2030 [28] and the Action for Malaria Investment 2016–2030 [29] reinforce the continued relevance of the Vivax Working Group agenda.

By 2015, the group had grown to include representatives from 18 national malaria control programmes, numerous *P. vivax* research partners, the WHO, as well as a variety of consortia and industry representatives.

### Membership of the working group

Members are drawn from two main groups: country partners with key responsibilities for delivering national malaria control activities, and academic and research partner institutions. Given the group’s technical focus, individuals with specific expertise and responsibilities for vivax malaria are nominated to represent either their country or institution, and over time the individuals have often changed. The primary aim of this collaborative interaction is to ensure that the working group conducts quality research with sound methodology, relevant to the needs of national programmes and regional elimination commitments.

The Working Group is supported by a coordinating team, including a programme coordinator and three part time research scientists. Additional logistical, advocacy and governance support is provided by the APMEN secretariat team. The work is funded by the Department of Foreign Affairs and Trade—Australian Aid Program (DFAT-APP, Australia) with additional support from the Bill and Melinda Gates Foundation (BMGF, USA) and financial and in-kind contributions from Partner Institutions and increasingly Country Partners. The Working Group members are listed in Additional file 1: Table S1 and the process of becoming a member is outlined in Additional file 2: Table S2.

### Approach to research and to influencing policy and practice

**Fig. 1:**
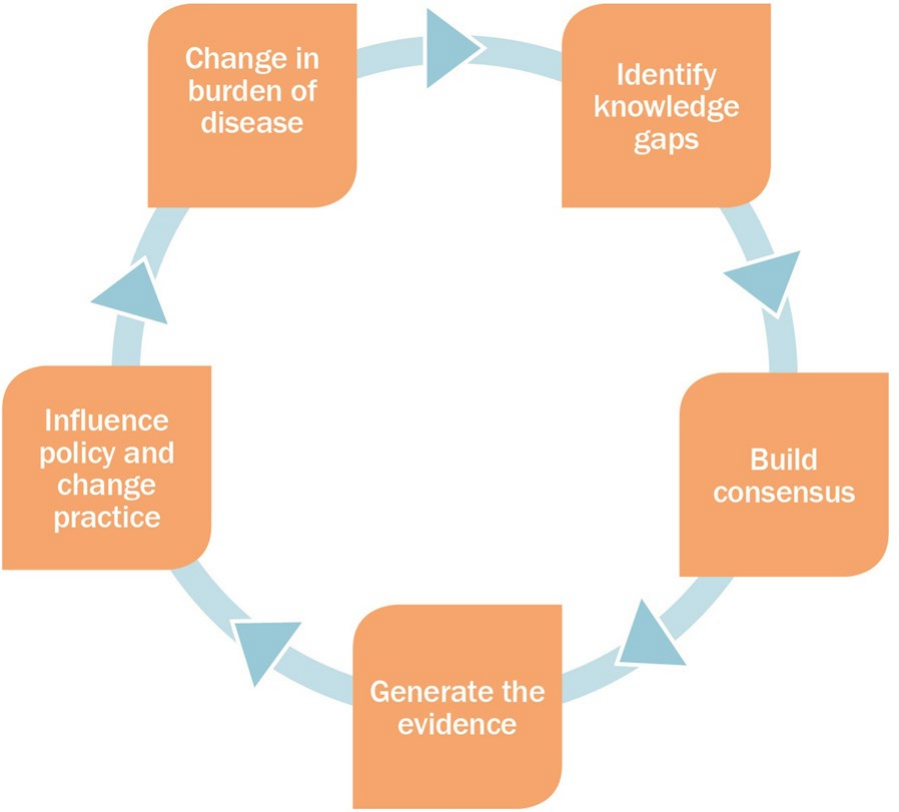
Cyclical works process of the VxWG. From: Asia Pacific Malaria Elimination Network. Targeting vivax malaria in the Asia Pacific 2009–2014. Brisbane, Australia. Asia Pacific Malaria Elimination Network; October 2014. P 15

The Working Group’s activities are geared to address key questions defined by the APMEN Country Partners and are closely aligned with the recent WHO technical brief on vivax malaria [30]. These activities can be directly relevant to operational research, but can also include more upstream research that will ultimately supply pathways to achieve these end goals. The Working Group’s programme responds to the needs of countries as they move through the different stages of elimination and is responsive to new developments and opportunities. The process can be summarized by the following four-phase cycle (Fig. 1):

1. The Working Group *Identifies key knowledge gaps* through systematic literature reviews and consultations with national control programmes and academic experts.
2. *Building consensus,* involves setting common agendas, developing projects and partnerships to address the key knowledge gaps which have been identified. Annual workshops, meetings and consultations are a critical part of this work.
3. The *Evidence* required to address the identified knowledge gaps is then generated through a coordinated programme funded directly by APMEN or by funding partners. The Working Group Coordinating Team provides technical support in the design, conduct and interpretation of small to medium sized research projects.
4. The fourth phase is the translation, where appropriate, of evidence into recommendations that *influence change in policy*.

### Identifying knowledge gaps

To date, the Working Group has conducted four systematic reviews. These include a review of the status of malaria research activities in member countries [31], the evidence on clinical efficacy of primaquine treatment options [32], key knowledge gaps for G6PD diagnostics [17] and the evidence for defining drug resistance in vivax malaria [16]. Key findings of those reviews are presented in Table 1.

**Table 1:** Key findings from the four APMEN Vivax Working Group literature reviews

Title	Main findings
Trends in malaria research in 11 Asian Pacific countries: an analysis of peer-reviewed publications over two decades	Between 1990 and 2009, there had been a significant decline in the proportion of malaria-related literature amongst all biomedical publications in the Asia-Pacific region [31].
Primaquine radical cure of *Plasmodium vivax*: a critical review of the literature	Treatment with low dose primaquine is not consistently effective in all areas. A higher dose of primaquine offers significant benefits in efficacy however these doses need to be confirmed in a range of endemic settings, and amongst high-risk patients. Multi-site trials are needed to assess higher doses of primaquine with a control arm, and careful and long-term patient follow up [32].
Review of key knowledge gaps in glucose-6-phosphate dehydrogenase deficiency detection with regard to the safe clinical deployment of 8-aminoquinoline treatment regimens: a workshop report	Improved diagnostics for G6PD deficiency are required to facilitate the broader, safe and effective use of primaquine. Current methods are impractical in areas with limited resources, and where most malaria patients live [17].
Global extent of chloroquine-resistant *Plasmodium vivax*: a systematic review and meta-analysis	Enhanced monitoring and better surveillance tools are needed to assess the burden of *P. vivax* malaria, identify areas of infection and drug resistance, and quantify changes in drug resistance patterns. Up to date information is critical to ensure optimal treatment recommendations [16].

### Building consensus

Consensus on research priorities is achieved through convened workshops and annual meetings. These forums include Working Group partners as well as other experts with specific expertise relevant to the themes of research. The thematic focus is on country partners needs to facilitate conducting locally relevant research. The process of common decision-making includes the participation of all members and is critical to the success of the group. All recommendations of the Working Group are reviewed by APMEN Advisory Board [33] before being tabled for formal voting endorsement by the Country Partners either at the Annual Business meeting or through electronic ballot. The board consists of three country partners and two partner institutions with voting rights; non-voting members are also present including representatives from WHO, the APMEN secretariat and funding bodies. Building consensus through exchange of ideas and sharing experience fosters collaboration and ensures that planned projects incorporate both innovative research approaches and the practical realities of implementation. Working within the APMEN structures and voting mechanisms ensures the evidence gathered is readily available and relevant to National Programmes. To date the network has convened eight workshops to promote dialogue and strive for consensus on approaches, methodologies and data sharing. Further details can be found in the Additional file 3: Table S3.

The process of engagement, collaboration and consensus is demonstrated in its application to parasite surveillance. Several countries in the later stages of elimination stressed the need to examine the potential of parasite genotyping to inform public policy interventions. In response, the first round of research projects funded by APMEN supported a large number of projects to genotype local *P. vivax* parasite populations in China, Indonesia, Malaysia, Republic of Korea, Sri Lanka and Bhutan. Although the projects had merit on their own, they were strengthened by the opportunity to compare genotyping datasets among the countries as well as within each individual study area. These comparisons required a consensus on genotyping methods, including internal standards, quality control and an appropriate platform for data sharing and standardized analysis. In response, a *P. vivax* genotyping workshop was held in 2011 in Sabah, Malaysia, to coordinate research activities amongst the Country Partners and develop consensus methods for genotyping. The partners agreed to use fragment size analyses of a set of 9 previously described short tandem repeat markers (MS1, MS5, MS8, MS10, MS12, MS16, MS20, pv3.27 and msp1F3) and to type a set of “standard” *P. vivax* samples to facilitate standardization of allele calling between different laboratories. A year later a second workshop was held in Incheon, Republic of Korea, to facilitate data sharing between sites and strengthen local capacity in data analysis. The VivaxGEN platform was developed to facilitate standardized allele calling and genotyping analysis that had been promoted at the Incheon workshop. The platform is currently being used as an internal resource for collaborating partners. Each partner retains ownership of their data including decisions regarding data accessibility via the vivaxGEN platform. Developments are underway to enable external researchers in the broader vivax research community to utilize the platform.

### Generating evidence

The Working Group endeavours to generate relevant evidence through the Country Partner Technical Development Programme (Additional file 4: Table S4). The allocated budget for this is targeted for junior to mid-level researchers to support research related activities and mentor future leaders in the field. Investments have been modest; most individual grants were below AUD$ 50,000 (USD$ 40,000). Between 2010 and 2014, a wide range of projects was funded on the three main research themes of surveillance, diagnosis, and treatment (Additional file 5: Table S5, Additional file 6: Table S6).

### Surveillance

Parasite surveillance activities range from quantification of parasite prevalence to optimizing the detection of asymptomatic and sub-patent infections, identifying major reservoirs of infection, detecting transmission hotspots and developing robust and informative approaches to *P. vivax* genotyping. Host surveillance focuses primarily on defining the prevalence of G6PD deficiency and variant types in individuals likely to be exposed to primaquine for radical cure in order to define the risk of drug-induced haemolysis during intensive control activities.

The parasite molecular surveillance programme has generated vigorous debate on the value of molecular approaches in informing malaria control activities. Whilst several countries in the earlier stages of malaria elimination envisage limited practical benefit of parasite genotyping, countries in the later stages are strongly in favour. Defining the parasite population genetic structure has potential to inform on transmission intensity [34, 35], geographical origin of infection (i.e. whether an infection was local or imported) [36, 37], and rapidly emerging clonal expansions associated with outbreaks [38–41]. Intensive research is underway to define molecular markers of *P. vivax* drug resistance to identify the emergence of drug resistant parasites [42] and prioritize sites for anti-malarial clinical trials. The outputs from the surveillance activities can provide evidence on drug efficacy to support review of treatment policies and revision if warranted. Innovations in molecular technologies mean that extensive data relevant to NMCPs can now be generated from small quantities of capillary blood, at continually declining costs.

Individual surveillance projects have limited potential to answer regional questions, pooling of multiple datasets is, therefore, important to better understand those. Many of the individual surveillance projects contribute to larger initiatives such as the Malaria Atlas Project (MAP) [43, 44], an APMEN partner which is collating global maps of malaria epidemiology [45, 46] and distribution of G6PDd [47] and variants [48]. MAP is a prime example of data sharing within and across national boundaries, with the VxWG providing a unique forum for mutual exchange of both data and methods, and the evaluation of preliminary mapping outputs by programme experts to ensure accuracy and programmatic value ahead of public release. The VxWG has facilitated a particularly successful collaboration with the Indonesian Ministry of Health [49]. For *P. vivax* molecular surveillance, the vivaxGEN platform, hosted by the Eijkman Institute for Molecular Biology and Menzies School of Health Research is another example of an online environment aimed at facilitating data sharing and comparative analysis [50]. In light of the challenges of highly mobile human populations, a unified approach to surveillance in the Asia-Pacific region is essential for successful elimination.

APMEN has funded 13 surveillance projects in five countries, including cross-sectional and community surveys adopting a variety of sampling and processing strategies (Additional file 5: Table S5). Table 2 describes examples of research outputs within the surveillance theme, taking into account the cyclical process of work.

**Table 2:** Example of the cyclical work process of the Vivax Working Group (surveillance theme**)**

Identify knowledge gaps	In the first round of the Country Partner Technical Development Programme (CPTDP) undertaken in 2011, 5 of the 11 successful grant recipients proposed *P. vivax* genotyping studies. These studies were funded in China, Indonesia, Malaysia, South Korea and Sri Lanka. It was apparent that several of the partners shared similar objectives and challenges in the design and interpretation of the proposed studies. The VxWG identified the need for new strategies to monitor the impact of interventions on the local parasite population, identify local hotspots of transmission, and detect rapidly emerging/outbreak strains (identified as the rapid expansion of infections with identical genotypes) early. In addition, several partners highlighted the need for molecular tools to confirm imported cases and determine their geographic origin; this cannot be addressed without an integrated, multi-country approach. The VxWG coordinating team therefore held a workshop to facilitate standardized methodologies.
Build consensus	In 2011, a *P. vivax* genotyping workshop was held in Sabah, Malaysia, to identify the markers that would best address the partners’ needs. A consensus panel of 9 short tandem repeat markers (STR) were selected to aid characterization of *P. vivax* within-host and population diversity as this reflects parasite transmission patterns. It was agreed that more information on the genome-wide diversity of parasites from different countries would be needed to identify optimal geographic markers. Nonetheless, the data generated on the tandem repeat markers would aid a feasibility analysis of the ability to distinguish infections from different countries using molecular methods. In 2012, a second *P. vivax* genotyping workshop was convened in Incheon, South Korea, to discuss approaches to facilitate data analysis and data sharing between the country partners. The partners agreed to a custom-made web-based platform (http://www.vivaxgen.menzies.edu.au) to facilitate these processes.
Gather the evidence	To date three of the CPTDP studies have been published. In the low endemic settings of Sabah, Malaysia, *P. vivax* genotyping demonstrated large clusters of identical strains emerging in the population [38]. This finding emphasized the critical need for parasite molecular surveillance to identify rapidly emerging strains (infections with identical genotype profiles at the 9 STRs) which might reflect highly adaptive strains such as drug resistant strains before they spread extensively: conventional surveillance methods do not address this challenge. In Central China, using samples collected in 2007–2010, low differentiation (frequent gene flow) was observed between parasite populations from Anhui Province, where *P. vivax* remains endemic, and neighbouring Jiangsu Province, where no local cases have been detected since 2012 [24]. This finding highlighted the risks of resurgence in highly mobile human populations. In Indonesia, genotyping demonstrated higher diversity lower differentiation in *P. vivax* versus *P. falciparum* [12], possibly reflecting greater potential for spread in *P. vivax*. The same trend was observed in the Solomon Islands in a study led by researchers at the Australian Army Malaria Institute (an APMEN Partner Institute) [10]. This finding further emphasized the adaptive potential of *P. vivax* and the need to maintain diligent surveillance in pre-elimination settings.
Change practice	Studies using the consensus markers are currently underway in a further nine countries, including 3 remaining CPTDP studies in Bhutan, South Korea and Sri Lanka.

### Diagnostics

Several point of care tests (PoCs) are available for malaria diagnosis [51, 52]. Older aldolase-based RDTs have low sensitivity for *P. vivax*, but newer pLDH-based RDTs have high (averaging 95 %) sensitivity for *P. vivax* comparable to that of HRP2 tests for *P. falciparum* [51]. However the test formats currently in use are not *P. vivax*-specific [51]. Key challenges include the lower parasite densities of *P. vivax* in asymptomatic versus symptomatic patients that challenge sensitive diagnosis, and the inability of current PoCs to identify hypnozoite carriage [53]. Hence, a significant proportion of *P. vivax* infected individuals go undetected and untreated [7]. In order to address this, the VxWG has supported the evaluation of available diagnostics [54]. Currently PCR assays are being developed to quantify sub-patent parasitaemia to identify major reservoirs of infection and focus elimination activities. The use of loop-mediated isothermal amplification (LAMP) has the potential to provide robust on-site diagnosis of parasitaemia in areas of low malaria endemicity, but this approach needs optimization for the detection of *P. vivax* infection [55].

The delivery of 8-aminoquinoline based radical cure of *P. vivax* is limited by the known risk of haemolysis in G6PD deficient individuals. The most widely used diagnostic assay is the fluorescent blood spot test [56], an assay which requires laboratory facilities and extended time to perform. Intensive research and development on quantitative and qualitative assays is underway to provide better point of care testing that will facilitate G6PD testing across the spectrum of clinical environments [17, 57–59]. The Working Group is collaborating with international experts and organizations such as PATH and the Foundation for Innovative New Diagnostics (FIND) to evaluate field-testing of novel point of care diagnostics. APMEN country partners were early evaluators of novel G6PD diagnostic tests and generated the evidence to inform a WHO evidence review group on G6PD testing for *P. vivax* management [60]. APMEN has funded four projects to evaluate rapid diagnostic tests for detection of *P. vivax* and G6PD deficiency [54, 55, 61] (Additional file 5: Table S5). Table 3 describes an example from the diagnostic theme.

**Table 3:** Example of the cyclical work process of the Vivax Working Group (diagnostic theme)

Identify knowledge gaps	In May 2012 the VxWG convened a workshop in Incheon, Korea to identify key knowledge gaps in the detection of G6PD deficiency [17]. The lack of robust evidence for the distribution of G6PD deficiency, and the relationship of G6PD deficiency and drug-induced haemolysis was discussed. Without reliable, convenient and sensitive point of care diagnostics, primaquine radical cure is often not prescribed, undermining *P. vivax* elimination efforts.
Build consensus	The group identified 10 key areas that are of highest research priorities, including: the mapping of G6PD deficiency, understanding drug induced haemolysis in G6PD deficient individuals, the identification of desirable test characteristics and the cost benefit analysis of routine G6PD testing.
Gather the evidence	Research projects in Cambodia, the Republic of Korea [54], Indonesia, the Philippines and China are currently being supported by the VxWG to address the identified research priorities. One of the hot spots of vivax malaria in Hainan, a province in southern China, is home to a number of ethnic minorities. In contrast Jiangsu province located in Central China is mostly inhabited by Han Chinese and has witnessed a great reduction of vivax malaria over the last decades and the complete elimination of *P. falciparum* since 1990. In 2013 the VxWG supported a cross sectional survey in Hainan and Jiangsu provinces to assess the population specific prevalence of G6PD deficiency. Participants were recruited among healthy individuals as well as febrile patients attending a health care facility. One drop of blood was collected from every participant together with information on the participants’ ethnic background. G6PD status was assessed at a reference centre applying a recently modified test assay (WST 8/1 PMS: methoxy PMS, Dojindo, Japan) for mass screening. Preliminary analysis indicated significant differences in the prevalence of G6PD deficiency between different geographic areas and ethnic groups ranging from close to 0 % to above 10 %.
Change practice	The marked differences in G6PD deficiency prevalence among different ethnic groups living in close geographic proximity highlights the need for routine G6PD testing as part of the national treatment guidelines for the treatment of vivax malaria. The investigators are currently evaluating point of care diagnostics suitable for this task.

### Treatment

The epicentre for drug resistant *P. vivax* is on the island of New Guinea, but evidence for declining chloroquine (CQ) efficacy has now been reported from across the vivax endemic world [62, 63]. Effective detection of drug resistant *P. vivax* parasites may help to combat this threat if timely changes in treatment policy can be implemented. Interpretation of the extent of CQ resistant *P. vivax* is challenging [42]. Declining anti-malarial efficacy manifests in prolonged clearance time of blood stage parasites and an increasing risk for late recurrent infections. The timing of late recurrences is dependent upon the pharmacology of the initial treatment regimen, the degree of drug resistance, and the level of host immunity. *Plasmodium vivax* treatment failure is, therefore, confounded not only by reinfection (in patients remaining within an area of ongoing transmission), but also by relapses, arising from later reactivation of the dormant liver stages. Current molecular techniques are unable to distinguish reliably between these events [64–66]. This has undermined the definition and diagnosis of CQ resistance and inhibited enthusiasm for generation of routine surveillance data. In the absence of evidence to the contrary, there is a tendency to assume that current anti-malarial treatment regimens continue to retain efficacy long after declining anti-malarial activity has begun to emerge. However, it is vital that the threat of CQ resistant *P. vivax* is acknowledged and greater resources are applied for developing standardized, validated and reproducible tools for its characterization.

The Working Group is supporting three clinical trials in Bhutan, Malaysia, and Vanuatu and the Solomon Islands (Additional file 5: Table S5). These trials are undertaken in regions with little or no prior experience in the conduct of clinical trials, and with limited resources. The aim is to build capacity to conduct valid clinical trials and apply standardization methodologies for the assessment of CQ resistant *P. vivax*. Table 4 describes an example from the treatment theme.

**Table 4:** Example of the cyclical work process of the Vivax Working Group (treatment theme)

Identify knowledge gaps	Two literature reviews were conducted on the treatment of *P. vivax*. The first review addressed the current knowledge gaps for the radical cure of *P. vivax* infection [32]. It revealed that treatment with low dose primaquine is not consistently effective in all geographical areas. The review further demonstrated that higher doses of primaquine offer significant benefits. However there are few data available and the review therefore concluded that these findings would need to be confirmed in a range of endemic settings, and amongst high-risk patients. The second review was conducted to define the extent and evidence regarding chloroquine resistant *P. vivax* [16]. The review suggests that chloroquine resistance has been underappreciated, with evidence for reduced susceptibility in many areas where vivax is endemic. It concluded that standardized methodologies and the development of novel tools are required for the more precise quantification of drug efficacy.
Build consensus	In 2011 the VxWG group convened in Jiangsu, China to discuss options for a multicentre trial assessing the efficacy of vivax treatment options. During the meeting the methodological challenges of crafting appropriate study designs were discussed, but no consensus for a common protocol was reached. Participants did, however, agree to fund 3 pilot studies with the aim of generating information that would guide the study design for larger multicentred trials.
Gather the evidence	One of the three clinical studies was performed in Sabah, Malaysia to assess efficacy of early parasite clearance of the current first line treatment, chloroquine compared with the artemisinin combination therapy (ACT) artesunate–mefloquine. The study followed patients up for 1 year to assess efficacy of primaquine in both study arms. Preliminary results show that high levels of chloroquine resistant *P. vivax* are now present in this region, and suggest that a change to a unified ACT protocol for all *Plasmodium* species may be warranted.
Change practice	Preliminary data from this study have been presented to the Malaysian Ministry of Health, and are currently under review.

### Influencing policy and change practise

The VxWG has become a recognized forum for the introduction and discussion of emerging tools and technology in the context of vivax elimination in the region, ensuring that the network partners can adopt appropriate innovations and technologies in a timely manner.

The Country Partner Technical Development Programme, which provides funding for small scale research projects has been a major achievement of the Working Group. These projects have been selected to address priority knowledge gaps identified by Country Partners as integral to the success of their malaria control programmes. At least as important, the projects also build vital local capacity that ensures country ownership and sustainability of local research activities. While some projects contribute to improving technical capacity and expertise, others directly impact policy and practice. One such example is the clinical study in Malaysia (Additional file 5: Table S5; Table 4) which assessed the efficacy of early parasite clearance of chloroquine, which is the current first line treatment and compared it with artesunate–mefloquine. Preliminary results show that high levels of chloroquine resistant *P. vivax* are now present in this region, and suggest that a change to a unified ACT protocol for all *Plasmodium* species may be warranted. The Malaysian Ministry of Health is currently reviewing their treatment guidelines for *P. vivax*.

Others projects have provide preliminary data that will help to secure additional funding from outside APMEN. For instance the study in Vanuatu (Additional file 5: Table S5) in which the investigators have generated pilot data for developing and attracting funding for a larger and more definitive clinical trial.

Some projects are now being scaled up to nation wide interventions. An example of this is a study in Bhutan which reviewed the introduction of an electronic surveillance system using mobile phones for disease mapping and early diagnosis (Additional file 5: Table S5). Based on the performance and user acceptability reported in the study the Bhutanese Vector-Borne Disease Control Programme incorporated this novel approach into its national surveillance system and is rolling it out to all areas at risk for malaria.

### Challenges of the VxWG

The VxWG has faced a number of significant challenges. The exchange of research data and methodologies requires group cohesion and established relationships between the APMEN Country Partners and Partner Institutions, this requires time and investment. This process was at various times interrupted by turnover of staff within the NMCP and new Country Partners or Partner Institutions joining the network. Critical elements for the success of voluntary working groups are participation and engagement. Due to conflicting demands within both the research Partner Institutions and NMCP (such as a dengue outbreak or staff promotion) certain countries were not as engaged as others and this resulted in longer timeframes to gain group consensus.

Cross border movement of people and parasites are a major issue in sustaining malaria transmission, hence malaria elimination is dependent upon control programmes working together across international boundaries on common research and technical issues. The expansion of the network is therefore critical to the success of the APMEN mission. Since 2009 APMEN has expanded from 10 founding countries to 18, as countries in the region made gains in controlling malaria and committed to national or subnational strategies for malaria elimination. Many of these new members represent malarious countries who contribute significantly to the burden of disease in the region such as India and Papua-New Guinea, who both joined APMEN in 2015.

The Country Partner Technical Development Programme was designed to provide the evidence to fill identified key knowledge gaps. However, the size of the funding as well as the time frame limited the scope of this endeavour, the translation of this knowledge into policy change and ultimately the impact on disease burden. Many country partners required significant assistance in increasing research capacity in order to participate in tackling the research questions of most importance to their programmes.

Finally all of the VxWG goals and indeed its very existence are dependent upon sustained funding. The uncertainty about future funding from 2016 and beyond has made programmatic and strategic planning beyond this time point very difficult.

## Discussion

Over the last 5 years the APMEN VxWG has established itself as a unique forum for national malaria control programme managers, researchers and collaborative partners to exchange ideas and discuss regionally relevant issues and develop innovative approaches that can be applied to vivax specific challenges. To the authors knowledge there is no other forum with a comparable ability to unite such diverse stakeholders working on *P. vivax*. Throughout the last few years substantial progress has been achieved in building relationships and trust within the members of the group, which are the foundation for the success of the programme. APMEN and its Working Group are now recognized as providing an important complementary role to the Asia Pacific Leaders Malaria Alliance (APLMA), WHO and other regional consortia working together towards the goal of malaria elimination. Evidence for optimizing malaria control activities is provided traditionally by research groups and institutions, but these are not necessarily focussed on the local public health priorities. Lack of communication between researchers and national malaria control programme officers can be a major obstacle to ensuring the translation of evidence into practice [67]. The diverse membership of the VxWG fosters open dialogue and the benefits offered and gained by each of the partners are considerable. The NMCPs can express and articulate the questions they need answered to effectively combat malaria and encourage the research community to address these. Researchers can assist NMCPs in conducting and analysing well-designed research and in return gain insights into programmatic priorities and align their own research agendas accordingly. In addition, the Working Group provides a platform for other consortia such as MAP, the World Wide Antimalarial Resistance Network (WWARN) and public private partnerships such as FIND, Medicines for Malaria Venture (MMV) and PATH to receive feedback on product development, revision of target product profiles and calls for data sharing for regional and global mapping.

The malaria elimination goal in the region is set for 2030. Political commitment is now being provided through the APLMA, a high level advocacy platform aimed at accelerating political commitment to achieve malaria elimination in the region. It is vital that such high level commitment is accompanied by innovative ideas and feasible local solutions that can be implemented on the ground. APMEN is in a prime position to bridge this gap. The structure and the approach of the VxWG provides a model to apply similar approaches to the broader agenda on malaria elimination and ultimately these could be extended to other diseases of public health importance.

## Additional files

10.1186/s12936-015-0958-y Vivax Working Group Members.
10.1186/s12936-015-0958-y APMEN Country Partner Membership.
10.1186/s12936-015-0958-y Overview of workshops within the APMEN Vivax Working Group.
10.1186/s12936-015-0958-y Mechanisms of the Country Partner Technical Development Programme.
10.1186/s12936-015-0958-y APMEN VxWG Research grants.
10.1186/s12936-015-0958-y List of Publications.
