# Supplementary material for: Targeting vivax malaria in the Asia Pacific: The Asia Pacific Malaria Elimination Network Vivax Working Group
Source: Malar J. 2015 Dec 1;14:484. doi: 10.1186/s12936-015-0958-y (PMC4667409; doi:10.1186/s12936-015-0958-y)
Supplement: Supplementary file 1 — 10.1186/s12936-015-0958-y Vivax Working Group Members. [file 12936_2015_958_MOESM1_ESM.pdf]

Supplementary Table 1: Vivax Working Group Members

Members are drawn from three separate groups within APMEN: Country Partners, Partner Institutions and the World Health Organization. Given the groups technical focus, individuals with specific expertise and responsibilities for vivax malaria are nominated to represent either their country or organisation.

In August 2015, the group includes representatives from the National Malaria Control Programs of:

- Bangladesh
- Bhutan
- Cambodia
- China
- Democratic People's Republic of Korea
- India
- Indonesia
- Lao People's Democratic Republic
- Malaysia
- Nepal
- Papua New Guinea
- Philippines
- Republic of Korea
- Solomon Islands
- Sri Lanka
- Thailand
- Vanuatu
- Vietnam

APMEN Partner Institutions are active participants in the Working Group. Of the 37 APMEN Partner Institutions, the following are actively involved in the Working Group:

- Australian Army Malaria Institute (AAMI), Brisbane, Australia
- Burnet Institute, Melbourne, Australia
- Centers for Disease Control and Prevention (CDC), Atlanta, US
- Foundation for Innovative New Diagnostics (FIND), Geneva, Switzerland
- Eijkman-Oxford Clinical Research Unit (EOCRU), Jakarta, Indonesia
- Institute of Medical Research, Goroka, Papua New Guinea
- International Centre for Diarrhoeal Disease Research (icddr, b) Bangladesh
- Karolinska Institute, Stockholm, Sweden
- London School of Hygiene & Tropical Medicine (LSHTM), London, UK
- Mahidol Vivax Research Unit (MVRU), Bangkok, Thailand
- Malaria Atlas Project (MAP), University of Oxford, Oxford, UK
- Malaria Research Centre, Universiti Malaysia Sarawak, Sarawak, Indonesia
- Medicines for Malaria Venture (MMV), Geneva, Switzerland
- Menzies School of Health Research, Darwin, Australia
- Pasteur Institute of Cambodia, Phnom Penh, Cambodia
- QIMR Berghofer Medical Research Institute (QIMR Berghofer), Brisbane, Australia
- Research Institute for Tropical Medicine (RITM), Muntinlupa City, Philippines
- Walter and Eliza Hall Institute of Medical Research (WEHI), Parkville, Australia
- WorldWide Antimalarial Resistance Network (WWARN), Oxford, UK
- World Health Organization (WHO): SEARO, New Delhi, India and WPRO, Manila, Philippines
