# Supplementary material for: Targeting vivax malaria in the Asia Pacific: The Asia Pacific Malaria Elimination Network Vivax Working Group
Source: Malar J. 2015 Dec 1;14:484. doi: 10.1186/s12936-015-0958-y (PMC4667409; doi:10.1186/s12936-015-0958-y)
Supplement: Supplementary file 2 — 10.1186/s12936-015-0958-y APMEN Country Partner Membership. [file 12936_2015_958_MOESM2_ESM.pdf]

Supplementary Table 2: APMEN Country Partner Membership

Country Partners are countries in the Asia Pacific Region, (countries defined within the World Health Organization's South-East Asia and Western Pacific Regions), which have declared (within their national malaria plans or similar national plan document) a goal of embarking on malaria elimination at the national or sub-national level; and in whose national jurisdiction APMEN participants may propose both research and elimination activities. New Country Partners should have already achieved significant progress toward elimination within their country's borders and should be able to show careful consideration of elimination feasibility. They must also be able to demonstrate significant political commitment to the goal of national elimination.

New Country Partner Approval Process:

- Prospective countries in the Asia Pacific region will write to the Chair of the APMEN Advisory Board, care of the APMEN Program Manager, with an expression of interest in joining APMEN
- The Chair of the Advisory Board will work with the Secretariat to provide further details of the process to the country requesting participant status
- An analysis of the implications for APMEN for work plan, budget and other resources will be developed by the Secretariat
- The applicant will be invited to attend the next Network Business and Technical meeting as an Observer, and be presented to the Country Partners as a future participant at that APMEN Network Meeting
- The analysis of the implications for the APMEN work plan, budget and other resources will be presented to the Country Partners at the time of submission of recommendation regarding the new country's application
- Nominated countries will be invited to become full participants upon approval by the Country Partners at the Annual Business meeting (through Country Partner vote). The process for joining APMEN for prospective Country Partners may also be undertaken via an out of session vote by the relevant APMEN participants, if required.
- It shall be the responsibility of the Secretariat to ensure that any nominees, candidates, or new participants are acquainted with the organisation's purposes, policies, and procedures.

More information can be found here:
http://static1.1.sqspcdn.com/static/f/471029/21049347/1353562554537/APMEN+Governance+Document+2012.pdf?token=oPAAdWzPEBSJCnPogi4bQ0csVH4%3D
