# Supplementary material for: Targeting vivax malaria in the Asia Pacific: The Asia Pacific Malaria Elimination Network Vivax Working Group
Source: Malar J. 2015 Dec 1;14:484. doi: 10.1186/s12936-015-0958-y (PMC4667409; doi:10.1186/s12936-015-0958-y)
Supplement: Supplementary file 3 — 10.1186/s12936-015-0958-y Overview of workshops within the APMEN Vivax Working Group. [file 12936_2015_958_MOESM3_ESM.docx]

**Supplementary Table 3:** Overview of workshops within the APMEN Vivax Working Group

| **Title** | **Date and location** | **Objectives** | **Outcome** |
| --- | --- | --- | --- |
| Research priorities | 2011 Malaysia | To define research priorities of the Working Group | Three areas of research have been defined: surveillance, diagnostics, treatment |
| *P. vivax* genotyping workshop I | 2011 Malaysia | i) Coordinate parasite genotyping research activities amongst country partners  ii) develop consensus methods for genotyping | Consensus genotyping methodology (currently used by partners institutes in Bhutan, China, Indonesia, Malaysia, Korea and Sri Lanka) |
| *P. vivax* genotyping workshop II | 2012 Korea | i) facilitate data sharing across the region  ii) strengthen local capacity | Consensus agreement reached in data analyses and sharing methods (using the vivaxGEN framework, [www.vivaxgen.menzies.edu.au](http://www.vivaxgen.menzies.edu.au)) |
| Planning a multicentre primaquine trial | 2011 China | i) discuss methodological issues around primaquine trials  ii) develop consensus protocols | It was agreed that pilot studies should be funded to provide more information regarding the challenges and to inform study design for larger trials. A total of 3 pilot studies have been funded. |
| G6PD deficiency detection | 2012 Korea | Review of knowledge gaps of G6PD diagnostics with regards to safe deployment of primaquine | Publication titled: Review of key knowledge gaps in glucose-6-phosphate dehydrogenase deficiency detection with regard to the safe clinical deployment of 8-aminoquinoline treatment regimens: a workshop report |
| Threat of antimalarial resistance to elimination | 2013 Indonesia | i)review consequences of drug resistance  ii)promote importance of monitoring drug resistance, using standardized methods and share results  iii)gather regional perspectives on surveillance of artemisinin resistance in *P. falciparum* and discuss ways to improve monitoring for chloroquine resistance in vivax | Identification of priorities:  Standardised methodologies needed (e.g. common protocol for vivax drug efficacy monitoring) |
| Analysis and Interpretation of Vivax Antimalarial Efficacy Studies | 2014 Cambodia | i)Exchange experience in setting up and conducting trials  ii)Consensus on statistical plan  iii)Preliminary analyses of data  iv)Plan for future studies | Preliminary data analyses performed for the three pilot studies.  Participants discussed the need to review clinical study design and objectives in the light of decreasing number of cases in all the participating countries. Suggestions included putting greater focus on case detection and management and shift the focus more to adherence and effectiveness studies than traditional efficacy trials |
| G6PD testing for radical cure | 2015 Philippines | 1. Understand current challenges and evidence gaps facing successful implementation of G6PD screening to support *P. vivax* control and elimination goals 2. Recommendations of scalable strategies to achieve radical cure of *P. vivax* with G6PD screening | The meeting was held shortly before the WHO officially recommended routine G6PD testing for radical cure.  A number of testing strategies around recently developed test assays were developed. Based on a series of country reports the feasibility and barriers to routine G6PD testing where discussed. The meeting concluded with a session on the economics of routine testing.  Publication titled: The challenges of introducing routine G6PD testing into radical cure: a workshop report. |
