# Supplementary material for: Targeting vivax malaria in the Asia Pacific: The Asia Pacific Malaria Elimination Network Vivax Working Group
Source: Malar J. 2015 Dec 1;14:484. doi: 10.1186/s12936-015-0958-y (PMC4667409; doi:10.1186/s12936-015-0958-y)
Supplement: Supplementary file 4 — 10.1186/s12936-015-0958-y Mechanisms of the Country Partner Technical Development Programme. [file 12936_2015_958_MOESM4_ESM.pdf]

Supplementary table 4: Mechanisms of the Country Partner Technical Development Program

**Background**	The *Country Partner Technical Development Program* aims to assist National Malaria Programs in the Asia Pacific by supporting small research projects that:<br><br>• build upon existing tools<br>• develop skills and expertise amongst junior to mid-level researchers<br>• promote collaborative approaches<br>• contribute to the development of new assessment, prevention and treatment tools<br><br>Projects funded through this program focus on country-specific components within the research priorities defined and revised at each annual APMEN meeting.<br>The *Country Partner Technical Development Program* is coordinated by the Vivax Working Group Coordinating Team based at Menzies School of Health Research. Menzies staff provides technical assistance, including proposal development, ethics review, field and laboratory implementation, data collection, analysis, reporting and publication.<br><br>Project proposals can be submitted from individuals or organisations from any of the APMEN County Partners but they must be endorsed by their respective country's National Malaria Control Program.<br><br>Ensuring research meets ethical standards is managed through review and project management processes.
**Review and Selection of Projects**	The *Country Partner Technical Development Program* review and awarding procedure contains five stages:<br><br>1. Selection of priorities and call for proposals<br>2. Submission<br>3. Review<br>4. Feedback<br>5. Selection by Review Panel<br><br>***Step 1: Selection of priorities and call for proposals***<br>Country Partners nominate and agree on priority areas for the *Country Partner Technical Development Program.* This is ratified at the Vivax Working Group Annual Meeting. Calls for proposal are made through APMEN and guidelines are made available on the APMEN website.<br><br>***Step 1: Submission***<br>Applicants submit a full project proposal.<br>All proposals must include an endorsement form the relevant National Malaria Control Program, a budget, project timeline and CV for all research applicants and co-investigators.<br><br>***Step 2: Review***<br>Proposals are reviewed by selected members of the APMEN Vivax Working Group. Proposals are peer reviewed by two people: one from an APMEN country partner and the other from a research institution or a member. Reviewers are allocated

proposals according to their expertise and providing no conflict of interest.

Applications are graded 1-5 by the Vivax Working Group reviewers based on the below criterion. Projects are assessed against the following criteria:

- Policy Relevance
- Significance
- Capacity Building
- Research design and methods
- Research team
- Communication and Engagement
- Budget
- Links to research previously supported by APMEN

The composite score from the reviewers are used by the research review panel to prioritise funding.
All reviewers are provided with criterion assessment guidelines that detail the expectations and standards to be met and standards of *confidentiality*, *anonymity* and *competing interests*.

Step 3: Feedback
Once proposal review has been completed, feedback is provided to applicants.
Based on the reviews, the VxWG Coordinating Team either assists applicants in the further development of proposals or sends proposals sent directly to the Review Panel (Step 4).
Projects deemed unsuitable by both reviewers and the VxWG Coordinating Team are informed that they have been unsuccessful.

Step 4: Selection by Review Panel
The Review Panel consider the reviewer's recommendations in light of available funds, distribution of projects within the network and make the final decisions.
The review panel endeavours to fund all projects that fulfil the criteria funding permitting. Where this is not possible because the budget is not sufficient the VxWG Coordinating team works with applicants /countries to develop proposals for external funding.

The review panel consider all elements of the proposed projects, including ethical considerations. The panel includes research specialists with extensive experience in the design and implementation of projects. The panel is comprised of international experts, with more than 20 years experience in the design and implementation of health research in the region and are based in institutions renowned for ethical conduct in indigenous communities and overseas.

If there is a lack of consensus amongst the research review panel, the APMEN Advisory Board may be consulted for advice and dispute resolution. Applicants are informed of the outcome of their applications by the Vivax Coordinating Team.

**Contractual Requirements**	Successful applicants will only be awarded Research Grants if they meet and agree to the following standards:<br><br>1. Secure relevant national ethical approvals, subcontracts cannot be signed without documented proof of ethics approval. Research conducted in more than one country requires ethical approval from all relevant ethic committees*.<br>2. Commit to providing interim and final narrative and financial reports. Receipts for expenses are to be kept and can be requested upon site monitoring visits.<br>3. Funding is allocated in 3 tranches, 50% when contract is signed, 40% upon approval of interim report and 10% upon approval of final report.<br><br>*Clinical Trials additional requirements*<br>1. Trials to be registered on an appropriate trials registry. These registries contain information regarding the design, conduct and administration of clinical trials. This helps ensure that research is accessible to all involved in health care and improves research transparency.<br>2. Trials are to be registered in both Australia, with the relevant authority and in the country they are being implement in. This ensures that trials to meet the Australian National Statement on Ethical Conduct in Human Research.<br>3. All projects are to maintain registration and approval from ethics board for the duration of the project.<br><br>* If a country does not have a National Ethics Committee then it is considered by the Menzies School of Health Research Ethics Committee http://www.menzies.edu.au/page/Research/Ethics_approval/
