# Supplementary material for: Targeting vivax malaria in the Asia Pacific: The Asia Pacific Malaria Elimination Network Vivax Working Group
Source: Malar J. 2015 Dec 1;14:484. doi: 10.1186/s12936-015-0958-y (PMC4667409; doi:10.1186/s12936-015-0958-y)
Supplement: Supplementary file 5 — 10.1186/s12936-015-0958-y APMEN VxWG Research grants. [file 12936_2015_958_MOESM5_ESM.pdf]

Supplementary Table 5: APMEN Vivax Working Group Research grants in alphabetical order

Bhutan	Molecular assessment of *P. vivax* transmission dynamics in Bhutan
Bhutan	Malaria elimination in Bhutan using mobile technology for disease mapping and early diagnosis
Bhutan	Parasite clearance and recurrence rates among patients with vivax malaria on chloroquine and primaquine therapy
Cambodia	Glucose-6-phosphate enzyme activity dynamics and G6PD qualitative test performance in G6PD deficient Cambodian patients undergoing weekly primaquine for acute uncomplicated vivax malaria
China	Improving the accuracy of *P. vivax* case reporting using molecular methods
China	Population screening for G6PD deficiency in China using an enzyme assay on filter paper dried bloodspots
China	Sero-epidemiological analysis for monitoring malaria elimination in China
China	Target malaria elimination intervention in China using spatial-temporal distribution analysis
Indonesia	Genetic diversity of *P. vivax* in Indonesia
Indonesia	Survey of G6PD variants on Sumba Island and development of PCR primers for each
Indonesia	Prevalence of G6PD deficient individuals in Bangka Island
Indonesia	Spatial analysis of the incidence of G6PD mutations in *Plasmodium vivax* infection in South Central Timor (SCT), East Nusa Tenggara (ENT) Province, Indonesia, 2013
Indonesia	Community survey on knowledge, attitude and practise of malaria intervention (diagnosis & treatment) for vivax malaria in Indonesia
Malaysia	Molecular genotyping of *P. vivax* in isolates in Sabah, Malaysia
Malaysia	Artesunate-mefloquine vs. chloroquine in patients with acute uncomplicated *P. vivax*: a randomized open label trial in Sabah, Malaysia
Philippines	A prevalence study of glucose-6-phosphate dehydrogenase deficiency and operational issues in applying the test in resource poor areas in the Philippines
Republic of Korea	Evaluation of sensitivity and specificity of RDTs using microscopy and PCR in vivax malaria detection
Sri Lanka	Dynamics of *Plasmodium vivax* parasite populations during malaria elimination efforts in Sri Lanka, with low transmission and unstable malaria
Sri Lanka	Assessing the prevalence of malaria parasites in displaced populations who have returned or resettled in the post conflict districts of Kilinochichi, Mullaithivu and Mannar in Sri Lanka
Vanuatu & Solomon Islands	Evaluation of safety and efficacy of two primaquine dosing regimens for the radical treatment of *Plasmodium vivax* malaria in Vanuatu and Solomon Islands
