# Supplementary material for: Targeting vivax malaria in the Asia Pacific: The Asia Pacific Malaria Elimination Network Vivax Working Group
Source: Malar J. 2015 Dec 1;14:484. doi: 10.1186/s12936-015-0958-y (PMC4667409; doi:10.1186/s12936-015-0958-y)
Supplement: Supplementary file 6 — 10.1186/s12936-015-0958-y List of Publications. [file 12936_2015_958_MOESM6_ESM.docx]

**Supplementary Table 6:** List of publications from the Vivax Working Group

| **Investigator** | **Project** | **Publication** |
| --- | --- | --- |
| Dr Ari Winasti | Survey of G6PDd Variants on Sumba Island and Development of PCR Primers for Each | Satyagraha AW, Sadhewa A, Baramuli V, Elvira R, Ridenour C, Elyazar I, Noviyanti R, Coutrier FN, Harahap AR, Baird JK. G6PD deficiency at Sumba in Eastern Indonesia is prevalent, diverse and severe: implications for primaquine therapy against relapsing vivax malaria. PLoS Negl Trop Dis. 2015 Mar 6;9(3):e0003602. doi: 10.1371/journal.pntd.0003602 |
| Dr Gawrie Galappaththy | Prevalence of malaria parasites in displaced population who have returned or resettled in the post-conflict districts of Kilinochichi, Mullativu and Mannar in Sri Lanka | Abeyasinghe RR, Galappaththy GN, Smith Gueye C, Kahn JG, Feachem RG. Malaria control and elimination in Sri Lanka: documenting progress and success factors in a conflict setting. PLoS One. 2012;7(8):e43162. doi: 10.1371/journal.pone.0043162 |
| Professor Gao Qi | Improving the accuracy of *P. vivax* case reporting using molecular methods | Tao ZY, Zhou HY, Xia H, Xu S, Zhu HW, Culleton RL, Han ET, Lu F, Fang Q, Gu YP, Liu YB, Zhu GD, Wang WM, Li JL, Cao J, Gao Q. Adaptation of a visualized loop-mediated isothermal amplification technique for field detection of *Plasmodium vivax* infection.  Parasit Vectors. 2011 Jun 21;4:115. doi: 10.1186/1756-3305-4-115 |
| Professor Gao Qi | Improving the accuracy of *P. vivax* case reporting using molecular methods | Liu Y, Auburn S, Cao J, Trimarsanto H, Zhou H, Gray KA, Clark TG, Price RN, Cheng Q, Huang R, Gao Q. Genetic diversity and population structure of *Plasmodium vivax* in Central China. Malar J. 2014 Jul 9;13:262. doi: 10.1186/1475-2875-13-262 |
| Dr Jung-Yeon Kim | Evaluation of *P. vivax* Diagnostic Methods, and Assessment of G6PD Prevalance and *P. vivax* Population Structure in Korea | Kim JY, Ji SY, Goo YK, Na BK, Pyo HJ, Lee HN, Lee J, Kim NH, von Seidlein L, Cheng Q, Cho SH, Lee WJ. Comparison of rapid diagnostic tests for the detection of *Plasmodium vivax* malaria in South Korea. PLoS One. 2013 May 7;8(5):e64353. doi: 10.1371/journal.pone.0064353. Print 2013. |
| Dr Jung-Yeon Kim | Evaluation of *P. vivax* Diagnostic Methods, and Assessment of G6PD Prevalence and *P. vivax* Population Structure in Korea | Goo YK, Ji SY, Shin HI, Moon JH, Cho SH, Lee WJ, Kim JY. First evaluation of glucose-6-phosphate dehydrogenase (G6PD) deficiency in vivax malaria endemic regions in the Republic of Korea. PLoS One. 2014 May 22;9(5):e97390. doi: 10.1371/journal.pone.0097390. |
| Dr Noor Rain Abdullah | Study of Drug sensitivity profile and Molecular Genotyping of *Plasmodium vivax* isolates in Sabah, Malaysia | Abdullah NR, Barber BE, William T, Norahmad NA, Satsu UR, Muniandy PK, Ismail Z, Grigg MJ, Jelip J, Piera K, von Seidlein L, Yeo TW, Anstey NM, Price RN, Auburn S. *Plasmodium vivax* population structure and transmission dynamics in Sabah Malaysia. PLoS One. 2013 Dec 17;8(12):e82553. doi: 10.1371/journal.pone.0082553 |
| Dr Rintis Noviyanti | Genetic diversity of *Plasmodium viva*x in Indonesia | Noviyanti R, Coutrier F, Utami RAS, Trimarsanto H, Tirta YK, Trianty L, Kusuma A, Sutanto I, Kosasih A, Kusriastuti R, et al: Contrasting Transmission Dynamics of Co-endemic *Plasmodium vivax* and *P. falciparum*: Implications for Malaria Control and Elimination, PLoS Negl Trop Dis  2015 May 7;9(5):e0003739. doi: 10.1371/journal.pntd.0003739 |
| Dr Preethi Randeniya | Dynamics of *Plasmodium vivax* parasite populations during malaria elimination efforts in Sri Lanka, with low transmission and unstable malaria. | Dias S, Wickramarachchi T, Sahabandu I, Escalante AA, Udagama PV. Population genetic structure of the *Plasmodium vivax* circumsporozoite protein (Pvcsp) in Sri Lanka. Gene. 2013 Apr 15;518(2):381-7. doi: 10.1016/j.gene.2013.01.003 |
| VxWG Working Group | Systematic Review – Status of malaria research in Asia Pacific? | Andersen F, Douglas NM, Bustos D, Galappaththy G, Qi G, Hsiang MS, Kusriastuti R, Mendis K, Taleo G, Whittaker M, Price RN, von Seidlein L. Trends in malaria research in 11 Asian Pacific countries: an analysis of peer-reviewed publications over two decades. Malar J. 2011 May 18;10:131. doi: 10.1186/1475-2875-10-131 |
| VxWG Working Group | Systematic Review - available evidence of clinical efficacy of primaquine treatment regimens? | John GK, Douglas NM, von Seidlein L, Nosten F, Baird JK, White NJ, Price RN. Primaquine radical cure of *Plasmodium vivax*: a critical review of the literature.  Malar J. 2012 Aug 17;11:280. doi: 10.1186/1475-2875-11-280 |
| VxWG Working Group | Systematic Review - What are key knowledge gaps in G6PD deficiency diagnostics? | von Seidlein L, Auburn S, Espino F, Shanks D, Cheng Q, McCarthy J, Baird K, Moyes C, Howes R, Ménard D, Bancone G, Winasti-Satyahraha A, Vestergaard LS, Green J, Domingo G, Yeung S, Price R. Review of key knowledge gaps in glucose-6-phosphate dehydrogenase deficiency detection with regard to the safe clinical deployment of 8-aminoquinoline treatment regimens: a workshop report. Malar J. 2013 Mar 27;12:112. doi: 10.1186/1475-2875-12-112. |
| VxWG Working Group | Systematic Review - What is the level and geographic range of drug resistance against *P. vivax* malaria? | Price RN, von Seidlein L, Valecha N, Nosten F, Baird JK, White NJ. Global extent of chloroquine-resistant *Plasmodium vivax*: a systematic review and meta-analysis.  Lancet Infect Dis. 2014 Oct;14(10):982-91. doi: 10.1016/S1473-3099(14)70855-2 |
| VxWG Working Group | The challenges of introducing routine G6PD testing into radical cure | Ley B, Luter N, Espino FE, Devine A, Kalnoky M, Lubell Y, Thriemer K, Baird JK, Poirot E, Conan N, Kheong CC, Dysoley L, Khan WA, Dion-Berboso AG, Bancone G, Hwang J, Kumar R, Price RN, von Seidlein L, Domingo GJ. The challenges of introducing routine G6PD testing into radical cure: a workshop report. Malar J. 2015 Sep 29;14:377. doi: 10.1186/s12936-015-0896-8. |
